# Application of Three-Dimensional Computed Tomography Improved the Interrater Reliability of the AO/OTA Classification Decision in a Patellar Fracture

**DOI:** 10.3390/jcm10153256

**Published:** 2021-07-23

**Authors:** Seong-Eun Byun, Oog-Jin Shon, Jae-Ang Sim, Yong-Bum Joo, Ji-Wan Kim, Young-Gon Na, Wonchul Choi

**Affiliations:** 1 Department of Orthopaedic Surgery, CHA Bundang Medical Center, CHA University, Seongnam 13497, Korea; sonofos@daum.net; 2 Department of Orthopedic Surgery, Yeungnam University Medical Center, Daegu 42415, Korea; ossoj@ynu.ac.kr; 3 Department of Orhopaedic Surgery, Gil Medical Center, Gachon University College of Medicine, Incheon 21565, Korea; sim_ja@hanmail.net; 4 Department of Orhopaedic Surgery, Chungnam National University Hospital, Daejeon 35015, Korea; ybjoo@cnu.ac.kr; 5 Department of Orthopaedic Surgery, Asan Medical Center, College of Medicine, University of Ulsan, Seoul 05505, Korea; bakpaker@hanmail.net; 6 Department of Orthopaedic Surgery, Seoul Segyero Hospital, Seoul 05790, Korea; orthonyg@gmail.com

**Keywords:** patella, patellar fracture, interrater reliability, AO/OTA classification, three-dimensional computed tomography, 3-D CT

## Abstract

We investigated whether interrater reliabilities of the AO/OTA classification of patellar fracture change with the imaging modalities applied, including plain radiography and two- and three-dimensional (2-D and 3-D) computed tomography (CT). Seven orthopedic specialists and four orthopedic residents completed a survey of 50 patellar fractures to classify the fractures according to the AO/OTA classification for patellar fractures. Initially, the survey was conducted using plain radiography only, then with 2-D CT introduced three weeks later and 3-D CT introduced six weeks later. Fleiss’ Kappa coefficients were calculated to determine interrater reliability. The overall interrater reliability of the AO/OTA classifications was 0.40 (95% CI, 0.38–0.42) with plain radiography only and 0.43 (95% CI, 0.41–0.45) with the addition of 2-D CT. With the addition of 3-D CT, the reliability was significantly improved to 0.54 (95% CI, 0.52–0.56). In specialists, interrater reliability of the classifications was moderate with all three imaging modalities. With the use of 3-D CT, interrater reliability of the classification was 0.53 (95% CI, 0.50–0.56), which was significantly higher than that with the use of 2-D CT (κ = 0.45; 95% CI, 0.42–0.48). In residents, interrater reliability of the classification was 0.30 (95% CI, 0.24–0.36) with plain radiography. The reliability improved to 0.49 (95% CI, 0.43–0.56) with the addition of 2-D CT, which was significantly higher than that with plain radiography only. The use of 3-D CT imaging improved interrater reliability of the classification. Therefore, surgeons, especially residents, may benefit from using 3-D CT imaging for classifying and planning the treatment of patellar fractures.

## 1. Introduction

The patella functions as a lever for the extensor mechanism of the knee joint, and it constitutes the patellofemoral joint with three-quarters of the posterior aspect covered by articular cartilage [1]. Therefore, more than 80% of patellar fractures are intraarticular fractures [2,3], and complications such as joint stiffness and postoperative arthritis can occur [4]. Anatomic reduction of the articular surface and rigid fixation are the treatment goals to prevent such complications.

For anatomic reduction and selection of the appropriate fixation method, precise evaluation and in-depth understanding of the fracture patterns are essential. The classification of the fractures plays an important role in the evaluation. For patellar fractures, the Arbeitsgemeinschaft für Osteosynthesefragen/Orthopaedic Trauma Association (AO/OTA) classification has been used and was recently revised [5]. However, the interrater reliability of the AO/OTA classification of patellar fractures were found to be only fair among trauma specialists, even with the use of two-dimensional (2-D) computed tomography (CT) [6].

Tension band wiring has been widely used as a fixation method for patellar fractures [7]. However, the relatively high failure rate [8,9] and increase in fractures that are not suitable for tension band wiring have resulted in an increase in the use of other fixation methods [2,10,11].

Three-dimensional (3-D) CT provides images of the patella that can be rotated and viewed from any direction. Using 3-D CT, surgeons can obtain an impression of the overall shape of the patellar fracture, including the articular surface. The use of 3-D CT in the evaluation of various intra-articular fractures has been increasing, and the role of 3-D CT in fracture classification and treatment planning for several intra-articular fractures has been evaluated [12,13,14,15]. However, to the best of our knowledge, no study has investigated the interrater reliabilities of the classification and treatment recommendations of patellar fractures using 3-D CT imaging.

Therefore, the purpose of this current study was to evaluate (1) the effect of the addition of 3-D CT on the interrater reliability of the AO/OTA classification for patellar fractures, and (2) the effect of imaging modality on the reliability according to the surgeons’ experience. The hypotheses of this study were (1) that each addition of 2-D and 3-D CT would increase the interrater reliability for the AO/OTA classification and (2) residents who have less experience interpreting plain radiographs compared to specialists will benefit more from the use of CT.

## 2. Materials and Methods

The present study was conducted after obtaining approval of the authors’ institutional review board. From 2013 to 2017, 72 cases with patellar fractures admitted to our hospital were initially enrolled for the analysis.

The inclusion criteria were patellar fractures that underwent preoperative imaging studies, including anteroposterior and lateral views of knee plain radiographs; 2-D CT in the axial, coronal, and sagittal planes; and 3-D CT within one week from initial trauma. The exclusion criteria were skeletally immature patients (two knees), patients with periprosthetic patellar fractures (three knees), and patients with a history of previous surgery on their patella (one knee). Cases with inadequate plain radiographs (four cases) and cases without CT images (12 cases) were also excluded. Finally, 50 patellar fractures were analyzed. The mean age of the patients was 52 years (range 22–81 years). There were 28 male patients and 22 female patients.

All CT images were created using an Optima CT 660 scanner (GE Healthcare, Milwaukee, WI, USA) with 2.0-mm thickness at the same hospital. Coronal and sagittal 2-D reconstructions were performed in the scanner, and volume-rendering 3-D image reconstruction was performed on a separate Advantage workstation (GE Healthcare, Milwaukee, WI, USA, version 4.2).

All identifiable data, including age and sex, were obscured for blinded evaluation. Eleven independent investigators, including seven orthopedic specialists who actively operate on patellar fractures in university hospitals, with a minimum of six years of experience (range, 6–18 years) and four orthopedic residents (4th–5th postgrad year) completed the survey.

**Figure 1:**
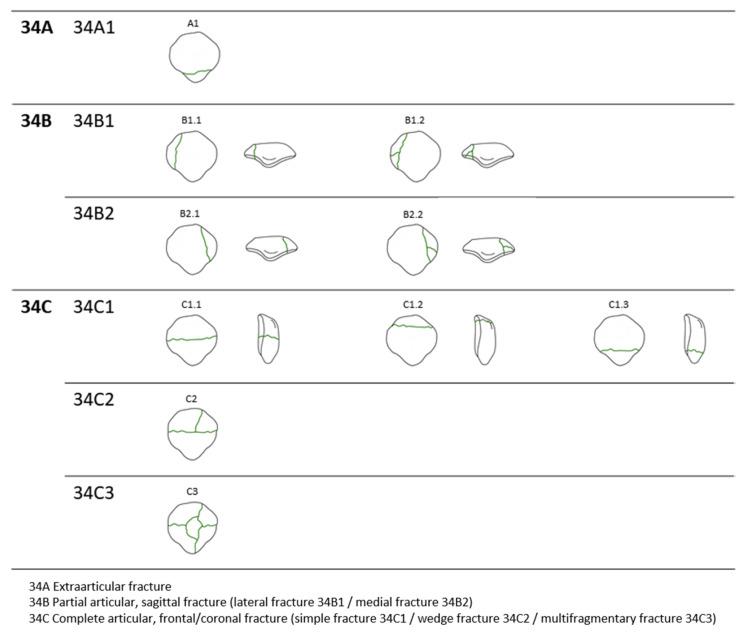
The AO/OTA classification of the patellar fracture.

The survey was conducted as follows: based on diagrams of each classification (Figure 1), respondents were asked to nominate the AO/OTA classification.

Three rounds of the survey were compared. Initially, the observers classified the fracture pattern based on plain radiography only. Three weeks later, the same survey was performed with plain radiography and 2-D CT. After an interval of three weeks, the observers conducted the survey with plain radiography, 2-D CT, and 3-D CT.

### Statistical Analysis

Fleiss’ kappa [16] coefficient was calculated for interrater reliability of fracture classification as the number of raters was more than two. In comparison of the two Fleiss’ kappa coefficients (interrater reliability), a statistically significant difference was considered in cases where the confidence intervals did not overlap.

According to Landis and Koch [17], kappa coefficients <0 indicate no agreement; 0.0–0.2, slight agreement; 0.21–0.4, fair agreement; 0.41–0.6, moderate agreement; 0.61–0.8, substantial agreement; and 0.81–1.0, almost perfect agreement. *p* < 0.05 reflected the chance that the interrater agreement was >0 (pure chance alone).

Sample size was calculated according to Walter et al. [18]. To determine the reliabilities of each subgroup, sample size was calculated based on the number of orthopedic residents (smaller subgroup). A minimum of 46 patellar fractures is required with supposing ρ0 = 0.3, ρ1 = 0.5, α = 0.05, β = 0.2. Based on this result and the exclusion criteria, 50 cases were enrolled for the survey.

All statistical analyses were performed using R Statistical Software version 3.3.0 (R Foundation for Statistical Computing, Vienna, Austria).

## 3. Results

**Figure 2:**
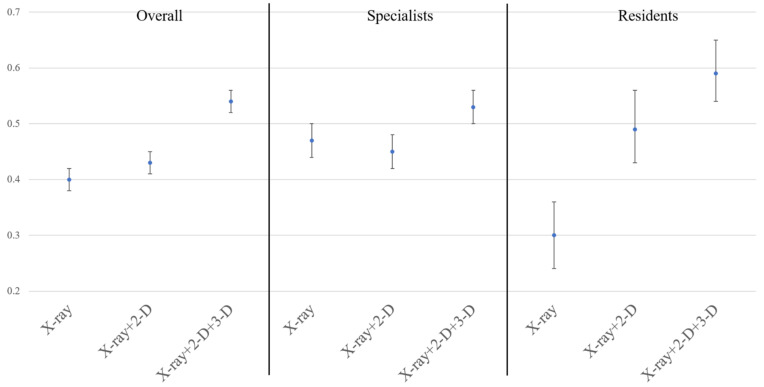
Interrater reliability of the AO/OTA classification. Blue dots indicate the calculated Fleiss kappa coefficient. Error bars indicate the 95% confidence interval. 2-D, two-dimensional computed tomography. 3-D, three-dimensional computed tomography.

The overall interrater reliability of the AO/OTA classification was fair (κ = 0.40; 95% confidence interval [CI], 0.38–0.42) with plain radiography only, and moderate (κ = 0.43; 95% CI, 0.41–0.45) with the addition of 2-D CT. With the addition of 3-D CT, interrater reliability of the AO/OTA classification was 0.54 (95% CI, 0.52–0.56), which was significantly higher than that of other modalities (Figure 2). The number of cases classified the same by all 11 participants was 5 with plain radiography only, 9 with the addition of 2-D CT, and 13 with the addition of 3-D CT.

Among specialists, interrater reliability of the classifications was moderate with all three imaging modalities. With the use of 3-D CT, interrater reliability of the classification was 0.53 (95% CI, 0.50–0.56), which was significantly higher than that with the use of 2-D CT (κ = 0.45; 95% CI, 0.42–0.48). Among residents, interrater reliability of the classification was 0.30 (95% CI, 0.24–0.36) with plain radiography. The reliability improved to 0.49 (95% CI, 0.43–0.56) with the addition of 2-D CT, which was significantly higher than that with plain radiography only. With the addition of 3-D CT, the reliability improved to 0.59 (95% CI, 0.54–0.65); however, the 95% CI overlapped with that of 2-D CT (Figure 2).

## 4. Discussion

The addition of 3-D CT significantly improved overall interrater reliabilities of the classification compared with plain radiography alone and plain radiography with 2-D CT. For specialists, the use of 3-D CT improved reliability for the classification compared with plain radiography alone and plain radiography with 2-D CT. For residents, interrater reliability was significantly improved with 2-D and 3-D CT compared with that with plain radiography only.

In the previous study by Lazaro et al. [6], only a ‘fair’ degree of interrater reliability of the AO/OTA classification was found, even with 2-D CT, among four specialists, and 2-D CT did not significantly improve the agreement. Similarly, our results showed that 2-D CT did not increase the interobserver reliability of all 11 participants, including four residents; however, the agreements among the residents did improve significantly with the addition of 2-D CT.

With only plain radiography, the interrater agreement for the classification of the residents was significantly lower than that of the specialists; however, with the addition of 2-D/3-D CT, no difference was found in the agreement between the specialists and the residents. Identifying the fracture line of the patella is sometimes difficult on the AP view, as the patella is overlapped with the femur, and the minimally displaced vertical fracture or secondary fracture line is often undetected. Previous studies have shown that CT is better for identifying inferior pole involvement, intra-articular step-offs, and gaps [3,6,19]. Therefore, residents, who are relatively less experienced in interpreting plain radiographs of patellar fractures, are more likely to benefit from using 2-D CT images, as per our hypothesis.

With the addition of 3-D CT, interrater reliability of the AO/OTA classification among all participants and specialists improved significantly. Considering the increased number of participants who selected the same classification with the use of 3-D CT and that most of these cases (11/13 cases) were classified as a C3 type, it can be interpreted that 3-D CT has an advantage in identifying the comminution in C-type fractures.

**Figure 3:**
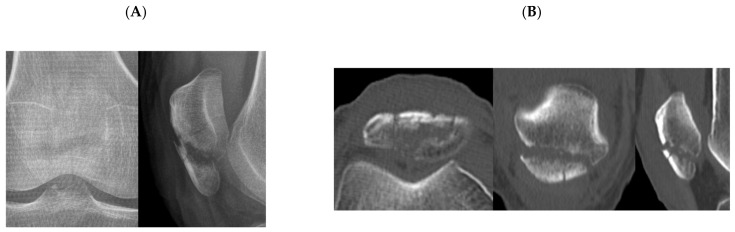

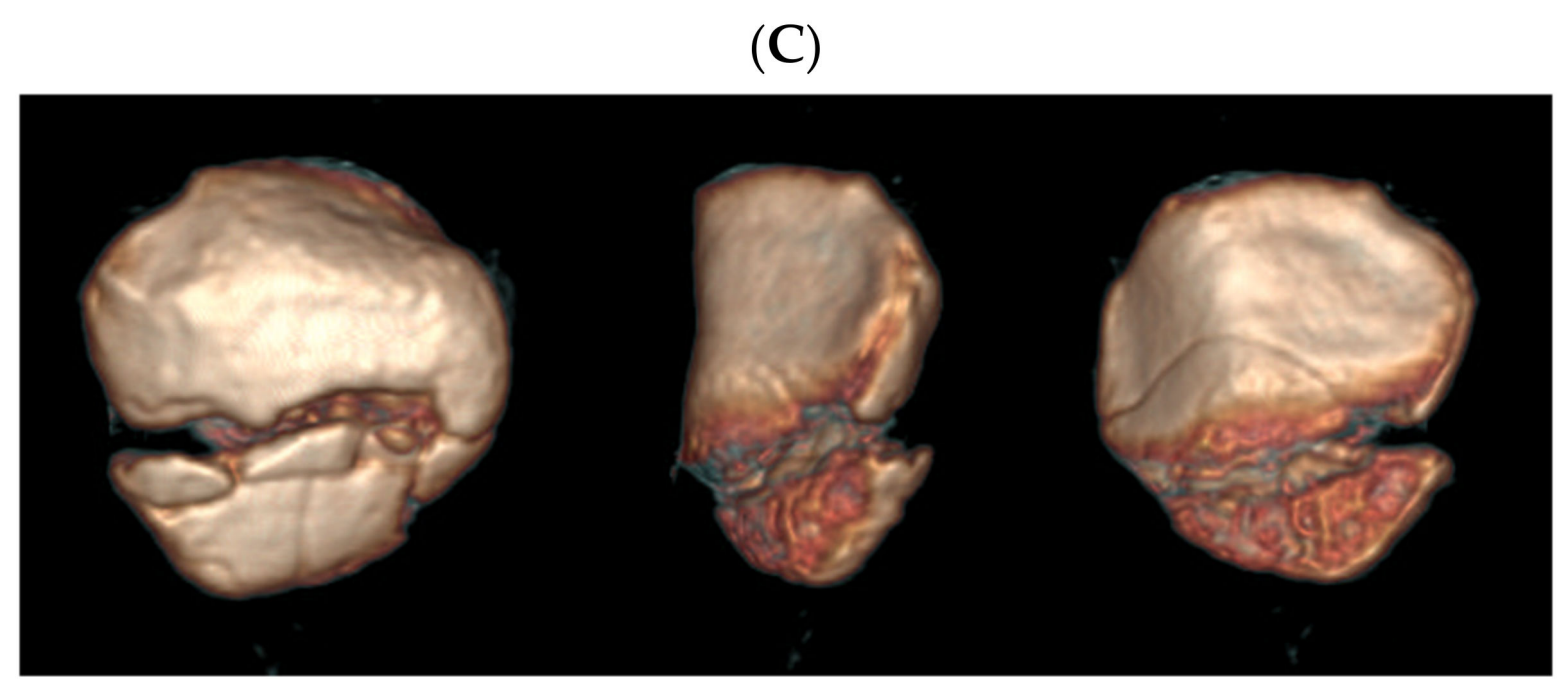
Plain radiographs (**A**), two-dimensional computed tomography (**B**), and three-dimensional computed tomography (**C**) of a patellar inferior pole fracture. In addition to the displaced extra-articular fracture, non-displaced linear articular fracture lines exist. The respondents chose either type A or C.

**Figure 4:**
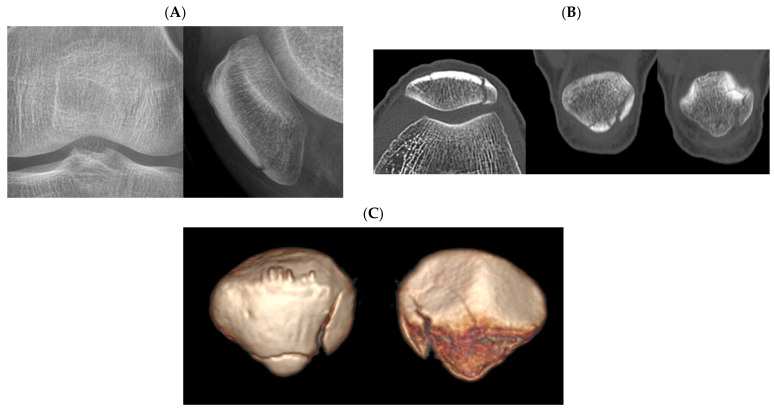
Plain radiographs (**A**), two-dimensional computed tomography (**B**), and three-dimensional computed tomography (**C**) of a patellar fracture with fracture lines of both horizontal and vertical fracture lines. The respondents chose either type A or B.

Fracture classification facilitates communication between surgeons, treatment planning, and prognosis prediction. Simplicity, as well as reliability, are important characteristics for a good fracture classification [20]. Therefore, fracture classification, including the current AO/OTA classification, may not be able to classify all fracture patterns. Lazaro et al. [6] suggested that the characteristics of the classification, not explicitly addressing the inferior pole comminution, may contribute to low reliability of AO/OTA classification. Inferior pole fractures occurring at the margin of the articular surface were also difficult to clearly distinguish between extra-articular (type A) and intra-articular fractures (type C) (Figure 3). There may be fractures in which two or more fracture types are combined. Due to the anatomical characteristics of the patella, where three-quarters of the posterior aspect is covered by articular cartilage, most fractures with complex fracture lines may be classified as type C3. However, there can be a fracture with extra-articular fracture and intra-articular fracture that cannot be classified as type C3. For example, a case showed both an extra-articular inferior pole fracture and partially articular vertical fracture lines. The respondents chose either type A or B and showed disagreement (Figure 4). Interrater reliability of AO/OTA classification may be affected by the above.

This study had several limitations. First, reliability, and not accuracy, was evaluated; therefore, it is necessary to be cautious while interpreting the increase and decrease in the kappa coefficient. Second, the study design was not appropriate to assess intra-rater reliability, since new image materials were added to each survey. Third, as the number of specialists and residents who participated in the survey was different, the overall result did not reflect the two groups equally. Therefore, the authors tried to minimize the bias by analyzing and presenting the results of specialists and residents separately.

## 5. Conclusions

The use of 3-D CT images improved interrater reliability of the AO/OTA classification. Residents are more likely to benefit from using CT images. Therefore, surgeons may benefit from using 3-D CT imaging for classifying and planning the treatment of patellar fractures.

## Data Availability

The data presented in this study are available on request from the corresponding author.
